# Runaway Competition: A Correction and Extension of Results for a Model of Competitive Helping

**DOI:** 10.1371/journal.pone.0164188

**Published:** 2016-10-06

**Authors:** Erin Wild, Monica Gabriela Cojocaru

**Affiliations:** Department of Mathematics & Statistics, University of Guelph, Guelph, Ontario, Canada; Southwest University, CHINA

## Abstract

We investigate and generalize an existing model of competitive helping within a biological market, first introduced for a population of competing individuals in which one individual provides help to all others; the rest compete for the help available from this individual by providing help themselves. Our generalized model comprises two strategies in which each individual of a specific type provides the same amount of help as all other individuals of that type. Each individual’s fitness function is dependent on this level of help, the cost of providing the help, and the fact that help is proportionally reciprocated by other individuals. Competitive helping occurs when individuals actively try to help more than other individuals. To assess the emergence of equilibrium help strategies as adopted by proportions of the population, we examine the competition over available help within two settings: replicator dynamics and agent-based numerical simulations. To move one step further in our generalization, we use the agent-based model to study the *N*-person competitive helping game, where all individuals in the population are heterogeneous with respect to help provided. Our results show that helping does not increase indefinitely with the population size, as concluded previously, and while there are some instances of an increase in help provided as a result of competition, this competition can be detrimental to all individuals and in most cases, one type simply gives up (thus evolving to a “no help” strategy). The degree to which an individual’s help is reciprocated by the others in the population has strong implications in the long-term behaviour of equilibrium help levels of types of individuals (and of individuals themselves); these equilibrium help levels diverge from existing conjectures in current literature. Lastly, small amounts of passively provided (costless) help results in runaway competition among all individuals.

## Introduction

Cooperation and the evolution of altruism between non-kin has been the focus of much research in evolutionary biology [1–5]. Natural selection results in inherent competition between individuals to survive and reproduce, and thus one would presume only selfish behaviour should be rewarded. Yet cooperation exists among and between many diverse species of animals including humans. Darwin noted it as a potential problem for his theory of natural selection [6].

Trivers first introduced the concept of reciprocal altruism as a mechanism to explain the evolution of cooperation [7]. Direct reciprocity relies on repeated interactions between two altruistic individuals in which one individual has a net cost less than the other’s net benefit [4, 8]. The net benefit to both individuals over a series of interactions is hence positive. Axelrod and Hamilton presented the Prisoner’s Dilemma as a game-theoretic model that could provide insight into the strategies leading to serial cooperation [1, 9]. Since then, researchers have presented alternative models that may explain a variety of other phenomena [5, 8, 10, 11].

Since costly help is a limited resource, an individual receiving attention and help from another results in less available for other individuals within the biological market [12, 13]. As a result, there is inherent competition to attract that attention, and by actively helping, individuals can perhaps increase their value in the eyes of others [13–15]. Competitive helping can occur when the best helpers receive more help than those helping less and when the benefit of receiving additional help outweighs the investment cost [13, 16]. More specifically, it occurs when individuals actively try to help more than everyone else. This sort of competitive altruism can serve as a courtship display, especially by males [17]. The potlatch, a gift-giving feast practiced by First Nations people along the Northwest Coast of North America, is another example of competitive altruism that sometimes cascaded into economic collapse [18].

In this paper, we generalize and correct the analysis of a competitive helping model started by Barclay in [13]. In this model, individuals divide their attention among other individuals and dispense help, similar to food dispensers in foraging models. The help that they produce is available to be claimed by those they pay attention to, and thus helpers will tend to receive more attention than non-helpers. In particular, the model comprises a population of individuals within a biological market confronted with the appearance of one individual who provides a benefit (i.e., help) to the other members of the population, who aren’t currently providing any help. The fitness function of this individual is dependent on the level of help it provides, the cost of providing this help, and the fact that its offer of help is proportionally reciprocated by the other individuals. Barclay concludes that the appearance of this individual will motivate the other individuals to offer some help as well and that competing over the help available in the population results in an increasing amount of help being provided [13].

In particular, we generalize the original model such that it explicitly comprises two types of individuals, each providing different levels of help. In order to assess the emergence of equilibrium help strategies as adopted by proportions of the population, we examine the competition over available help within two settings: replicator dynamics and agent-based numerical simulations. Replicator dynamics [19] let us determine equilibrium values for the proportions of both types whose help strategies we fix; while type-based numerical simulations reveal behaviour and equilibrium values of help provided by fixed fractions of the population allowed to evolve their help incrementally over time. Finally, we examine individual-based numerical simulations to gain insight into equilibrium help levels of individuals who are heterogenous in their levels of help provided.

We investigate the conditions under which the strategy of each type persists. We find that by correcting the analysis and generalizing both the theoretical and computational framework for the study of the proposed biological market with competition over help, the previously conjectured hypotheses do not hold, while other interesting implications emerge. To the former point, the original analysis in [13] leads to a series of conjectures: that equilibrium levels of help (as in, optimal levels that maximize the payoff of an individual) depend on the population size, *N*, and the degree of having the help reciprocated, *z*; and that offering help should be an invading strategy (over a market of individuals who do not offer any help). Our analysis shows that both of these conclusions are not supported by the full mathematical investigation of the model. Most interestingly, we find that competition can be detrimental to those involved and small amounts of passively provided help results in runaway competition among all individuals.

## Analysis

### Model generalization

Outlined below is an overview and generalization of the model by Barclay [13] where the interaction of individuals in the population embodies a non-cooperative game in which individuals compete for available help. Within the biological market, a fixed number of competing individuals, *N*, is assumed and the population is partitioned into two types: those who produce help at a rate of *h*_*p*_ and those who produce help at a rate of *h*_*m*_, which Barclay [13] called *regular* and *mutant* individuals respectively. The proportion of the population of the first type is denoted *S*_*p*_ and the proportion of the latter type *S*_*m*_, with *S*_*p*_ + *S*_*m*_ = 1.

If we have a population in which only one individual of a certain type exists, then from their perspective the available help to be competed over is (*N* − 1)*h*_*p*_, and at any time, they are competing with *N* − 2 individuals over the attention of each of the *N* − 1 other individuals. As a result, Barclay [13] defines the total amount of help received, *r*, by a single individual in a population consisting of only individuals of the other type as:

$$\begin{array}{c} r=\left(N-1\right)h_{p}\frac{h_{m}}{h_{m}+\left(N-2\right)h_{p}}. \end{array}$$ (1)

We generalize this for populations consisting of two types with proportions of any size. An individual receives a portion of each other individuals’ help according to the proportion that they provide, as is the case in Barclay’s model [13]. Given the fractions of each type in the population, total help produced is *S*_*p*_*Nh*_*p*_ + *S*_*m*_*Nh*_*m*_. Thus, for an arbitrary individual *i* (of either type) producing help at a rate of *h*_*i*_, the amount of help they receive is denoted by *r*_*i*_ and defined as the sum of help received from all other individuals:

$$\begin{array}{c} r_{i}=\sum_{j\neq i}^{N}h_{j}\frac{h_{i}}{S_{p}Nh_{p}+S_{m}Nh_{m}-h_{j}}. \end{array}$$ (2)

We note for clarification purposes that *h*_*i*_ and *h*_*j*_ will be equal to one of *h*_*p*_ or *h*_*m*_ depending on the type of individuals *i* and *j*, respectively (i.e., if individual *i* is of type *p*, then *h*_*i*_ will be equal to *h*_*p*_).

The help received, *r*_*p*_ and *r*_*m*_, can then be rewritten in terms of *S*_*p*_ and *S*_*m*_ as:

$$\begin{array}{ccc} r_{p} & = & \frac{\left(S_{p}N-1\right){h_{p}}^{2}}{\left(S_{p}N-1\right)h_{p}+S_{m}Nh_{m}}+\frac{S_{m}Nh_{m}h_{p}}{S_{p}Nh_{p}+\left(S_{m}N-1\right)h_{m}},\\ r_{m} & = & \frac{S_{p}Nh_{p}h_{m}}{\left(S_{p}N-1\right)h_{p}+S_{m}Nh_{m}}+\frac{\left(S_{m}N-1\right){h_{m}}^{2}}{S_{p}Nh_{p}+\left(S_{m}N-1\right)h_{m}}. \end{array}$$ (3)

Eq (1), as well as the generalized *r* in Eq (3), assumes that each individual receives help in perfect proportion to the relative amount they provide. This is inspired by existing literature in operant conditioning on the matching law [20, 21], which predicts that there is a quantitative relationship between the relative rates of response and relative rates of reinforcement. In this model, each individual is a *source* with an associated *benefit* conferred upon others for associating with them. To account for the fact that perfect proportional matching rarely occurs in nature, Barclay [13] introduces *z* ≥ 0 to represent “the degree to which one receives help in proportion to the relative amount of help one provides,” based on deviations called undermatching, overmatching, and bias described by Baum [22, 23] and Wearden and Burgess [24]. The expectation is that when *z* = 0, individuals receive equal help regardless of how much they provide; when *z* = 1, perfect proportional matching occurs; and when *z* = ∞, the most generous individual receives all of the help produced (i.e., winner-takes-all). We will show that the latter case does not completely hold as expected given the way the model was constructed.

The assumption that individuals can sometimes produce help at no cost to themselves is also added, with *k* representing the amount of such passive help provided. Barclay assumes that all individuals provide the same amount of passive help, for model tractability purposes, and we follow suit. The generalized equation for *r*_*i*_ then becomes:

$$\begin{array}{c} r_{i}=\sum_{j\neq i}^{N}\frac{\left(h_{j}+k\right){\left(h_{i}+k\right)}^{z}}{S_{p}N{\left(h_{p}+k\right)}^{z}+S_{m}N{\left(h_{m}+k\right)}^{z}-{\left(h_{j}+k\right)}^{z}}, \end{array}$$ (4)

with,

$$\begin{array}{ccc} r_{p} & = & \frac{\left(S_{p}N-1\right)\left(h_{p}+k\right){\left(h_{p}+k\right)}^{z}}{\left(S_{p}N-1\right){\left(h_{p}+k\right)}^{z}+S_{m}N{\left(h_{m}+k\right)}^{z}}+\frac{S_{m}N\left(h_{m}+k\right){\left(h_{p}+k\right)}^{z}}{S_{p}N{\left(h_{p}+k\right)}^{z}+\left(S_{m}N-1\right){\left(h_{m}+k\right)}^{z}},\\ r_{m} & = & \frac{S_{p}N\left(h_{p}+k\right){\left(h_{m}+k\right)}^{z}}{\left(S_{p}N-1\right){\left(h_{p}+k\right)}^{z}+S_{m}N{\left(h_{m}+k\right)}^{z}}+\frac{\left(S_{m}N-1\right)\left(h_{m}+k\right){\left(h_{m}+k\right)}^{z}}{S_{p}N{\left(h_{p}+k\right)}^{z}+\left(S_{m}N-1\right){\left(h_{m}+k\right)}^{z}}. \end{array}$$ (5)

The total fitness payoff, *W*, to a single individual is defined as the difference between the fitness benefits and the cost of producing help [13]. Barclay [13] uses a diminishing marginal returns function rather than a constant cost/benefit ratio. This can be applied to an individual of either type in a similar manner as above:

$$\begin{array}{ccc} W_{p} & = & \frac{mr_{p}}{mx+r_{p}}-h_{p},\\ W_{m} & = & \frac{mr_{m}}{mx+r_{m}}-h_{m}, \end{array}$$ (6)

where *m* is the maximum fitness benefit from receiving help and *x* determines the rate of diminishing marginal returns (see Table 1 for a list of all variables).

**Table 1:** List of variables used in the model.

Variable	Definition
*N*	Number of individuals in the biological market.
*h*_*p*_	Level of (costly) help provided by type-p individuals.
*h*_*m*_	Level of (costly) help provided by type-m individuals.
*r*_*i*_	Help received by an individual of type *i* ∈ {*p*, *m*}.
*S*_*i*_	Proportion of the population of type *i* ∈ {*p*, *m*}.
*W*_*i*_	Payoff received by an individual of type *i* ∈ {*p*, *m*}.
*z*	Degree of matching.
*m*	Maximum fitness benefits from receiving help.
*x*	Variable affecting the rate of diminishing marginal returns.
*k*	Level of passive (costless) help provided by all individuals.

The conclusions in [13] are that: i) competitive helping increases with the population size, *N*, and ii) with the degree of matching, *z*; iii) equilibrium help levels are highest at low-intermediate values of *x*; iv) some passively-produced help, *k*, is necessary in the absence of any mutant helper for competitive helping to invade, but once it has, passive helping is no longer necessary.

Our results, stemming from our analyses below, show that the above conclusions do not hold as stated: i) helping does not increase indefinitely with the population size; ii) we find a different level of help that maximizes payoff in the case examined by [13], and in general, equilibrium help values scale with the maximum fitness benefit, *m*. The level of help provided in order to stay competitive increases as *z* increases, but the actual number of individuals competing decreases. iii) Moreover, equilibrium levels of help are highest at low-intermediate values of *x* when *z* ≤ 1, but as *z* increases, equilibrium help is highest at increasing values of *x*; iii) passive helping can force competition between individuals even when it is disadvantageous for everyone, but without it, many individuals will provide no help at all.

### Replicator dynamics model

Evolutionary stable states are found among stationary points of a replicator dynamics model [19]. Therefore, in this section, we formulate and analyze such a game that reflects our extension of the competitive helping model introduced above.

With the generalized equations for *r* and *W* given in Eqs (5) and (6), replicator equations are defined below in order to describe the change in the proportions of the population as a function of each type’s payoff over time.

$$\begin{array}{ccc} {\dot{S}}_{p} & = & S_{p}\left(W_{p}-\left(S_{p}W_{p}+S_{m}W_{m}\right)\right),\\ {\dot{S}}_{m} & = & S_{m}\left(W_{m}-\left(S_{p}W_{p}+S_{m}W_{m}\right)\right) \end{array}$$ (7)

Equating the replicator equations in Eq (7) to zero and solving for equilibria may provide insight into the existence and behaviour of steady states of the types and their help levels.

$$\begin{array}{c} 0={\dot{S}}_{m}=S_{m}\left(W_{m}-S_{p}W_{p}-S_{m}W_{m}\right)=S_{m}\left(\left(1-S_{m}\right)W_{m}-S_{p}W_{p}\right) \end{array}$$ (8)

Recall that *S*_*p*_ = 1 − *S*_*m*_, which gives 0 = *S*_*m*_(1 − *S*_*m*_)(*W*_*m*_ − *W*_*p*_). This implies that the population is in equilibrium when either all individuals are of one type with none of the other (i.e., *S*_*m*_ = 0, *S*_*p*_ = 1; or *S*_*m*_ = 1, *S*_*p*_ = 0), or the payoffs for individuals of both types are the same (*W*_*m*_ = *W*_*p*_). If the payoff to an individual is the same regardless of their strategy, there is no incentive to switch to the other strategy.

$$\begin{array}{c} W_{m}-W_{p}=0⟺\frac{m^{2}x\left(r_{m}-r_{p}\right)}{\left(mx+r_{m}\right)\left(mx+r_{p}\right)}-h_{m}+h_{p}=0 \end{array}$$ (9)

**Lemma 1**. Eq (9) *has solutions of the form*: (*h*_*p*_, *h*_*m*_ = *h*_*p*_), (*h*_*p*_, *h*_*m*_) = (0, *m* − *mx*), (*h*_*p*_, *h*_*m*_) = (*m* − *mx*, 0).

*Proof*. Assume that the level of active help provided is the same for both types (*h*_*m*_ = *h*_*p*_). Then the help received by both types is also equal (*r*_*m*_ = *r*_*p*_), and thus *h*_*m*_ = *h*_*p*_ leads to a solution of Eq (9). Now, assume that one of *h*_*m*_ or *h*_*p*_ is 0. By direct computation, we obtain that the other is equal to *m* − *mx*. In this case, the type’s fitness benefits and costs are the same and thus the payoff is zero for both types with no incentive to switch.

We can find the level of active help that maximizes the payoff in each of these cases by finding the derivative of *W*_*i*_ with respect to *h*_*i*_, setting it to zero, and solving for *h*_*i*_.

*Remark* If the proportions of types in the population are not of equal size, *h*_*p*_ is not equal to *h*_*m*_ and cannot be assumed. Since the model in [13] is formulated with *S*_*p*_ ≠ *S*_*m*_, the equation for h* found in that paper is incorrect.

**Lemma 2**. *Given the case in which* *h*_*p*_ = *h*_*m*_, *we first find that* *S*_*m*_ *must equal* *S*_*p*_. *From here, the optimal help provided can be derived as*:

$$\begin{array}{c} h^{*}=(-x+\sqrt{x}\cdot \frac{\sqrt{\frac{1}{2}\left(N-2\right)((z+1)N-1)}}{N-1})m-k \end{array}$$ (10)

*Remark* We note that the limit of Eq (10) as *N* becomes sufficiently large does not, in fact, continue to depend on *N* and thus helping does not increase indefinitely with population size, which seems to contradict the title claim in [13].

**Lemma 3**. *When* *S*_*p*_ = 1 *or* *S*_*m*_ = 1, *the help provided by the corresponding type* (*h*_*p*_ or *h*_*m*_ *respectively) which maximizes the payoff is simply* $h^{*}=\left(-x+\sqrt{x}\right)m-k$ *and does not depend on the degree of matching*, *z*, *or the population size*, *N*.

**Fig 1:**
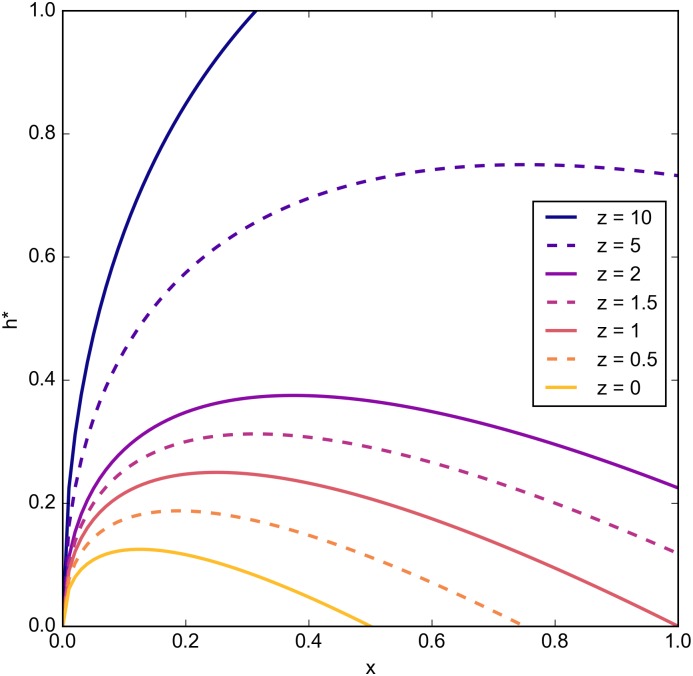
Optimal help values, h* Eq (10), with respect to *x* ∈ [0, 1] for various *z* values. We use the default values of *m* = 1, *k* = 0, and *N* sufficiently large, and we note that increasing values of *m* only scale the resulting curves, without changing their qualitative behaviour. Similarly, increasing values of *k* simply directly reduce the amount of optimal active help provided.

As *z* increases, we also note the increasing value of *x* for which equilibrium help is highest (Fig 1).

*Remark* By evaluating the Jacobian of the replicator equations at the equilibrium points found above, we can determine the sign of the eigenvalues and thus the stability of these equilibria. For the equilibrium points in which one of *S*_*p*_ or *S*_*m*_ is equal to 1 while the other is 0 (and assuming *k* = 0), we find one eigenvalue of multiplicity 2: h*(h* + *mx* − *m*)/(h* + *mx*). This eigenvalue is negative (and thus asymptotically stable) when $h^{*}=\left(-x+\sqrt{x}\right)m<m\left(1-x\right)$, which holds true for all values of *m* and *x*. The third equilibrium results in the same eigenvalue above with multiplicity one as well as an eigenvalue of zero. This indicates that there should be a line of equilibria along *S*_*p*_ = *S*_*m*_, however, we require that *S*_*p*_ + *S*_*m*_ = 1, and thus should be stable at *S*_*p*_ = *S*_*m*_ = 0.5 provided h* < *m*(1 − *x*). In this case, we must use h* from Eq (10) and we find that the eigenvalue is negative only when *z* < 2/*x*−1, which is also the threshold for nonnegative payoffs.

Further equilibria, assuming unknown values for both *h*_*p*_ and *h*_*m*_, not necessarily equal, are investigated in the next section via simulations since the derivative of the payoff function is too complicated to extract meaning from when *S*_*p*_ ≠ *S*_*m*_. Additionally, we examine the equilibrium trajectories of the population over time while studying their sensitivity to various degrees of matching, *z* > 0.

### Experimental design

The basic experiment uses a population comprising two types of fixed proportions; all individuals of the same type are considered homogeneous. The help provided by individuals evolves over time as each type attempts to maximize their payoff. At each time step, both types simultaneously mutate the current help they provide (incrementally) and calculate the expected payoff from making this change given their knowledge of the help provided by the other type during the previous time step. If the expected payoff is greater than their current payoff, they update the help provided to this new value, otherwise their help remains unchanged.

The simulation is repeated for constant values of *S*_*m*_ from 0 to 1 at increments of 0.1, with *S*_*p*_ = 1 − *S*_*m*_. The aggregate population size in these simulations is chosen to be 100000 with *m* = 1, *x* = 0.5, *k* = 0, and a mutation rate of 0.001 as default values. Since the proportions of each type are fixed over time in these simulations, we investigate the resulting equilibrium in terms of *h*_*m*_ and *h*_*p*_ and the associated payoffs, as well as the effect of the degree of matching, *z*. Further simulations allow for the proportion to change over time in addition to the help provided by each such type. At each time step, there is a chance that some percentage of individuals will realize they would likely receive a higher payoff by switching to the opposite type and thus they do so. The likelihood of switching is proportional to the difference in current payoffs between types.

Additional experiments were performed using a heterogeneous population of 200 individuals in which each agent controls the amount of help they personally provide. We also investigate the model’s sensitivity to positive levels of passive help, *k*, provided by individuals in the population.

## Results

**Fig 2:**
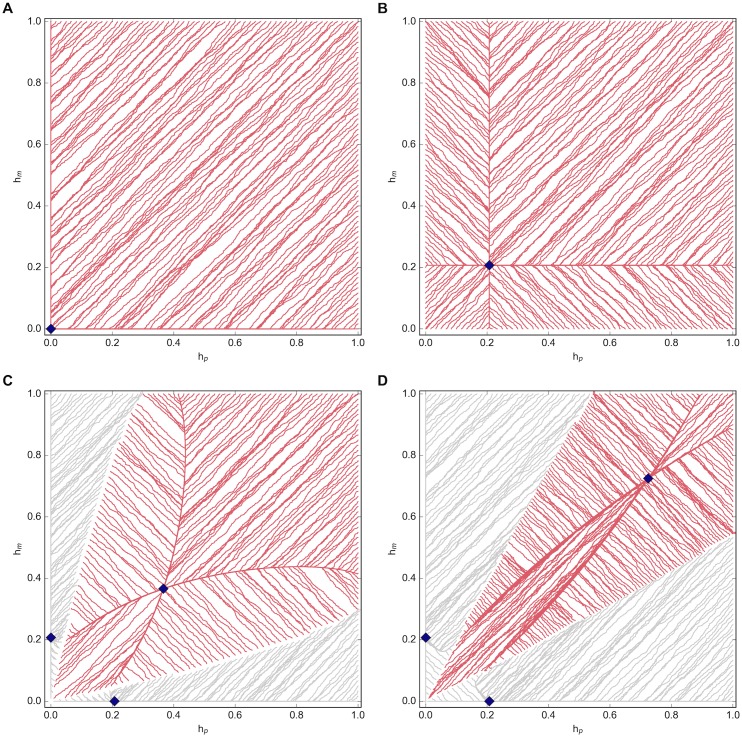
Equilibrium help trajectories with fixed proportions. (a) *z* = 0; (b) *z* = 1; (c) *z* = 2; (d) *z* = 5 when *S*_*m*_ = *S*_*p*_ = 0.5. The initial choice of *h*_*m*_ and *h*_*p*_ dictates the direction of evolution (as shown by the red and grey lines) towards the resulting observed equilibria (denoted by ♦). In particular, the basins of attraction for each equilibrium can be seen, with the red lines corresponding to the competitive helping equilibrium and the grey lines corresponding to one type providing no help.

The trajectory towards the equilibrium help values of each group of simulations is partially shown in Fig 2a-2d for values of *z* ∈ {0, 1, 2, 5} and when *S*_*m*_ = *S*_*p*_ = 0.5. In particular, these plots show the evolution of help over time, illuminating the basins of attraction for the equilibria. We note that the fixed proportions have some effect on the boundaries of these basins of attraction, while the initial choice of *h*_*m*_ and *h*_*p*_ dictates the direction of evolution and the resulting observed equilibrium.

When *z* = 0, there is only one equilibrium point and it shifts from $\left(h_{p}^{*},h_{m}^{*}\right)=\left(\left(-x+\sqrt{x}\right)m-k,0\right)$ to (0, 0) as *S*_*m*_ increases from 0 to 0.5 (Fig 2a shows *S*_*m*_ = 0.5). A further shift to $\left(0,\left(-x+\sqrt{x}\right)m-k\right)$ would occur if *S*_*m*_ increased to 1. For *z* = 1, the single equilibrium remains constant at (h*, h*), unaffected by the proportions of each type in the population (Fig 2b). As *z* increases beyond 1, a second and third equilibrium emerge in which one type provides help at a rate of $(-x+\sqrt{x})m-k$ while the other type provides no help (Fig 2c and 2d). These two points remain constant regardless of the values for *S*_*m*_ and *S*_*p*_, though the basins of attraction shift slightly. The main competitive helping equilibrium which is characterized by (h*, h*) at *S*_*m*_ = *S*_*p*_ = 0.5 clearly shifts towards the upper right of the plot as *z* increases, while the basin of attraction for this particular equilibrium shrinks. We can then say that as *z* increases so too does the amount of helping that each type provides in order to stay competitive, though the frequency of such competition decreases. The initial values of *h*_*m*_ and *h*_*p*_ must be close enough for competition to arise, otherwise one type will cease providing help altogether. Though not shown in these plots, we also note that competition results in an increasingly worse (and eventually negative) payoff.

**Fig 3:**
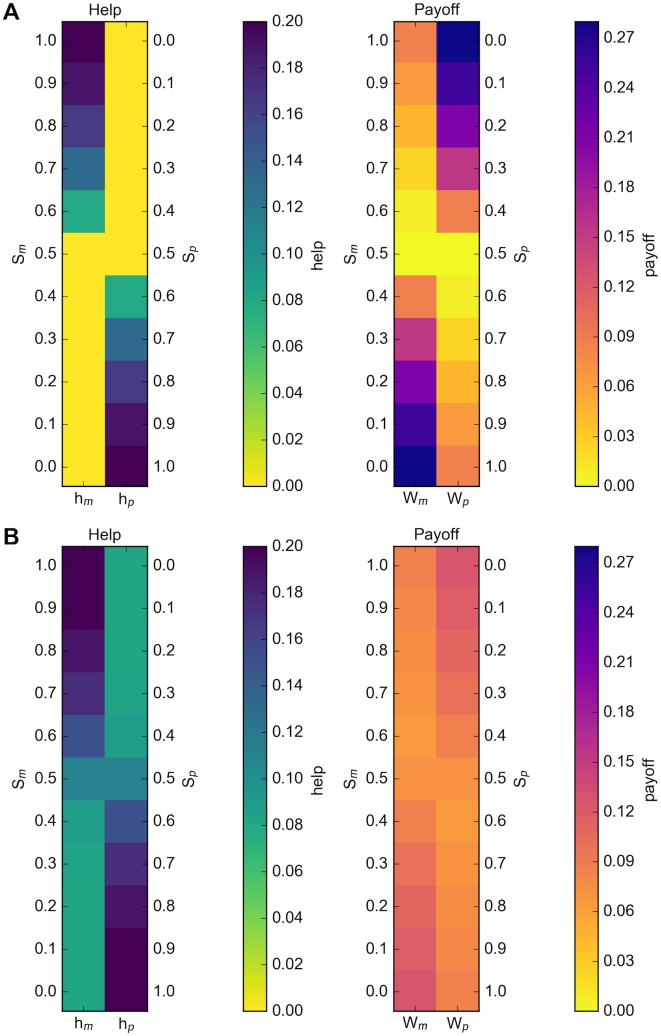
Equilibrium help and payoff values for fixed proportions. (a) *z* = 0; (b) *z* = 0.5 for various fixed proportions of *S*_*m*_ and *S*_*p*_.

The heatmaps in Fig 3a and 3b further illustrate that *h*_*p*_ equals *h*_*m*_ only when *S*_*p*_ also equals *S*_*m*_. When the proportions of each type in the population are uneven we see that the larger proportion provides more help but receives a smaller payoff than the other. Individuals would have an incentive to switch types if this were allowed and we would arrive at an equilibrium of *S*_*p*_ = *S*_*m*_ = 0.5 even though the payoff is lower for everyone.

**Fig 4:**
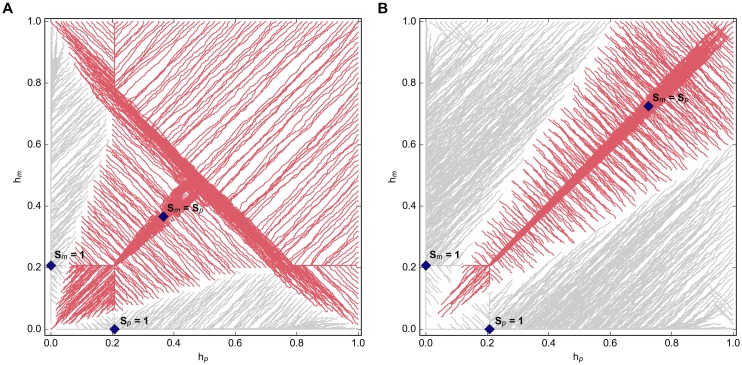
Equilibrium help trajectories with evolving proportions. (a) *z* = 2; (b) *z* = 5 for evolving proportions of *S*_*m*_ and *S*_*p*_.

By allowing individuals to move from one type to the other over the course of the simulation, we can identify which, if any, of the proportional splits represent stable equilibria. We expect to see *S*_*m*_ = 1 and *S*_*p*_ = 1 (from the replicator dynamics model) and *S*_*m*_ = *S*_*p*_ = 0.5 (from the analysis of the heatmaps in Fig 3a and 3b) emerge as equilibrium points, depending on the value of *z*. The trajectories are distorted when the proportions, *S*_*m*_ and *S*_*p*_, are also allowed to evolve, but the equilibrium help values remain the same (Fig 4a and 4b). We note that in these cases the equilibrium represented by *h*_*m*_ = *h*_*p*_ corresponds directly to an *S*_*m*_ = *S*_*p*_ result; while the equilibrium represented by *h*_*m*_ = 0, *h*_*p*_ = h* coincides with *S*_*m*_ = 0, *S*_*p*_ = 1 (and vice versa).

**Fig 5:**
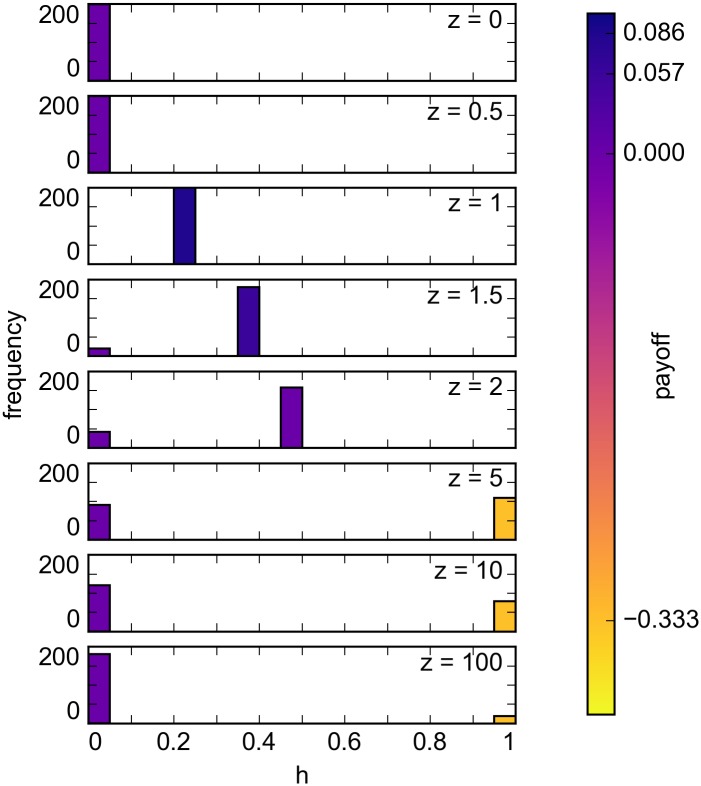
Frequency of equilibrium help provided in heterogeneous populations. The resulting payoff for providing help in each equilibrium is shown and we can see that payoff decreases as competitive helping among some individuals increases the amount of help they provide. We also observe that less individuals participate in competitive helping as *z* increases.

For heterogeneous populations, and particularly in the case of larger *z*, we found that competitive helping results in an increasing level of help provided at an increasingly smaller and eventually negative payoff (Fig 5). We also note that the act of competitive helping decreases as *z* increases, with a greater number of individuals providing no help instead.

**Fig 6:**
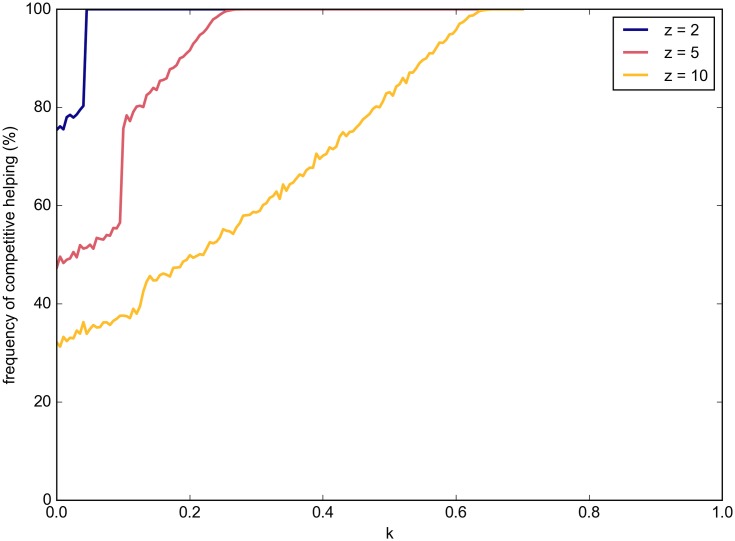
Frequency of resulting competitive helping equilibrium with increasing passive help provided. Small amounts of passive help, *k*, force competition between individuals.

However, when a small amount of passive help is provided, the equilibrium represented by competitive helping (*h*_*m*_ = *h*_*p*_) becomes the only one observed over any number of replicates of the simulation (Fig 6). The threshold of passive helping required for this equilibrium to dominate is very low for values of *z* near 1 and increases slowly as *z* increases. It is interesting to note that the payoff for competitive helping is still negative near and slightly above this threshold and doesn’t become positive unless more passive help is provided.

*Remark* An evolutionary stable strategy (ESS) is such that if adopted by all individuals in the population it cannot be invaded by any alternative strategy through natural selection [19, 25]. Let *E*(*S*, [*T*]) represent the payoff for playing strategy *S* against the rest of the population playing strategy *T*. The strategy of providing no help (i.e., *h* = 0) is an ESS when *E*(0, [0]) > *E*(*H*, [0]), which holds true for all values of *H* because *E*(*H*, [0]) = −*H* and 0 > −*H*. An individual’s payoff for providing help is only greater than zero if another individual is also providing help; this shows that there is a critical threshold of two for invasion of helping within a population of non-helpers.

## Conclusion

In simulating the competitive helping model with two types, we are able to shed some light on the behaviour hinted at in the original analysis [13]. We highlighted the main conjectures of the original model above and below we summarize our own findings in terms of these conjectures.

**Help provided does not increase indefinitely as populations become larger.** For populations of sufficient size, which we found to be as little as 200 individuals, the resulting equilibrium does not depend on population size. For smaller populations, the individuals fall short of converging on the optimal help to provide that was calculated above.

**Equilibrium help values are largest at low-intermediate values of *x* for *z* ≤ 1 only.** As *z* increases, equilibrium help is higher for larger values of *x* (Fig 1).

**Critical threshold of two for invasion.** Helping can invade non-helping only if two or more individuals start helping during the same time step. If only one individual tries to help, their payoff will be negative instead of zero if they did not help. With the lag in information about the amount of others’ help provided in the simulations above, it would be impossible for helping to invade non-helping without some sort of collusion between individuals.

**Passive help reduces active help at equilibrium.** When individuals produce some amount of passive help, the amount of active help provided is reduced by this amount (see Eq (10)).

**Bifurcation as a result of degree of matching, *z*.** The value of the equilibrium represented by *h*_*m*_ = *h*_*p*_ increases as *z* increases, while the proportion of simulations resulting in this particular equilibrium decreases and we begin to see equilibria in which one type stops helping altogether. The type that doesn’t give up provides help at the optimal value, $h^{*}=\left(-x+\sqrt{x}\right)m$ (Fig 2a-2d).

**Competitive helping is disadvantageous to all individuals.** As *z* increases, the value of the equilibrium represented by *h*_*m*_ = *h*_*p*_ increases beyond the optimal value and results in increasingly negative payoffs for both types (Fig 5). The idea of a ‘winner-take-all’ biological market as *z* approaches infinity is hence false in this model.

**Passive helping forces competitive helping when *z* > 1.** Small amounts of passive help results in the equilibrium represented by competitive helping (*h*_*m*_ = *h*_*p*_) being the only one observed over any number of replicates of the simulation (Fig 6).

Finally, we note that when proportions of each type in the population are able to evolve alongside help, the two equilibria represented by one of the types ceasing to provide help are actually characterized by no individuals of that type remaining (*S*_*m*_ = 0 or *S*_*p*_ = 0, when *h*_*m*_ = 0 or *h*_*p*_ = 0, respectively). As a result, all individuals are in fact providing equal and optimal help.

The main purpose of this research was to provide an in-depth analysis of an existing model of competitive helping, presumably derived from a biological perspective. In particular, we were interested in the emergence of equilibria through simulations which could not be found mathematically, and furthermore, the influence of parameters on the observed equilibrium help values. In looking to more clearly analyze the effects of the degree of matching, we showed that the results do not seem to correspond with the desired objective in constructing of the model (i.e., winner does not take all). Most interestingly, we found that competition is increasingly detrimental to those involved and when passive help (e.g. byproduct benefits, mutualisms) is provided such competition is ingrained in everyone.

## Supporting Information

S1 Text Main Java code used for simulations. (JAVA)

S2 Text Java class code used for simulations. Population.java defines classes used to create a population of individuals in the main simulation code.
(JAVA)

S3 Text Java class code used for simulations. PopulationGroups.java defines classes used to create a population of groups of individuals in the main simulation code.
(JAVA)

S4 Text MersenneTwister Java code used for simulations. The Mersenne Twister [26] is an exceptionally high-quality, fast random number generator; coded in Java by Sean Luke [27].
(JAVA)

## References

[1] Axelrod R. The Evolution of Cooperation. Basic Books; 1984.

[2] Sachs JL, Mueller UG, Wilcox TP, Bull JJ. The evolution of cooperation. Q Rev Biol. 2004;79(2):135–160. 10.1086/383541 15232949

[3] Vogel G. The evolution of the Golden Rule. Science. 2004;303:1128–1131. 10.1126/science.303.5661.1128 14976292

[4] Nowak MA. Five rules for the evolution of cooperation. Science. 2006;314:1560–1563. 10.1126/science.1133755 17158317 PMC3279745

[5] Clutton-Brock T. Cooperation between non-kin in animal societies. Nature. 2009;462:51–57. 10.1038/nature08366 19890322

[6] Darwin C. On the Origin of Species. John Murray; 1859.

[7] Trivers RL. The evolution of reciprocal altruism. Q Rev Biol. 1971;46:35–37. 10.1086/406755

[8] Noë R. A veto game played by baboons: a challenge to the use of the Prisoner’s Dilemma as a paradigm for reciprocity and cooperation. Anim Behav. 1990;39:78–90. 10.1016/S0003-3472(05)80728-6

[9] Axelrod R, Hamilton WD. The evolution of cooperation. Science. 1981;211:1390–1396. 10.1126/science.7466396 7466396

[10] Connor RC. Altruism among non-relatives: alternatives to the ‘Prisoner’s Dilemma’. Trends Ecol Evol. 1995;10(2):84–85. 10.1016/S0169-5347(00)88988-0 21236964

[11] Tooby J, Cosmides L. Friendship and the Banker’s Paradox: other pathways to the evolution of adaptations for altruism. P Brit Acad. 1996;88:119–143.

[12] Seyfarth RM. A model of social grooming among adult female monkeys. J Theor Biol. 1977;65(4):671–698. 10.1016/0022-5193(77)90015-7 406485

[13] Barclay P. Competitive helping increases with the size of biological markets and invades defection. J Theor Biol. 2011;281:47–55. 10.1016/j.jtbi.2011.04.023 21550351

[14] Noë R, Hammerstein P. Biological markets: supply and demand determine the effect of partner choice in cooperation, mutualism and mating. Behav Ecol Sociobiol. 1994;35:1–11. 10.1007/BF00167053

[15] Roberts G. Competitive altruism: from reciprocity to the handicap principle. Proc Biol Sci. 1998;265(1394):427–431. 10.1098/rspb.1998.0312

[16] Barclay P, Willer R. Partner choice creates competitive altruism in humans. Proc R Soc B: Biol Sci. 2007;274:749–753. 10.1098/rspb.2006.0209 PMC2197220 17255001

[17] Barclay P. Altruism as a courtship display: some effects of third-party generosity on audience perceptions. Br J Psychology. 2010;101:123–135. 10.1348/000712609X435733 19397845

[18] Harkin ME. Potlatch in Anthropology In: Smelser NJ, Baltes PB, editors. International Encyclopedia of the Social & Behavioral Sciences. vol. 17 Oxford: Pergamon Press; 2015 p. 11885–11889.

[19] Taylor P, Jonker L. Evolutionary stable strategies and game dynamics. Math Biosci. 1978;40:145–156. 10.1016/0025-5564(78)90077-9

[20] Williams BA. Reinforcement, choice, and response strength In: Atkinson RC, editor. Steven’s Handbook of Experimental Psychology, Vol. 2: Learning and Cognition. 2nd ed John Wiley & Sons; 1988 p. 167–244.

[21] Domjan M, Burkhard B. The Principles of Learning and Behavior. 3rd ed Brooks/Cole Publishing; 1993.

[22] Baum WM. Matching, undermatching, and overmatching in studies of choice. J Exp Anal Behav. 1979;32:269–281. 10.1901/jeab.1979.32-269 501274 PMC1332902

[23] Baum WM. Optimization and the matching law as accounts of instrumental behavior. J Exp Anal Behav. 1981;36:387–403. 10.1901/jeab.1981.36-387 16812255 PMC1333108

[24] Wearden JH, Burgess IS. Matching since Baum. J Exp Anal Behav. 1982;38:339–348. 10.1901/jeab.1982.38-339 7175431 PMC1347873

[25] Maynard Smith J, Price GR. The logic of animal conflict. Nature. 1973;246:15–18. 10.1038/246015a0

[26] Matsumoto M, Nishimura T. Mersenne Twister: A 623-Dimensionally Equidistributed Uniform Pseudo-Random Number Generator. ACM Transactions on Modeling and Computer Simulation. 1998;8(1). 10.1145/272991.272995

[27] Luke S. The Mersenne Twister in Java; 2004. https://cs.gmu.edu/~sean/research/

